# 

**Published:** 1948

**Authors:** 


### The Bristol Medico-Chirurgical Journal

A JOURNAL OF THE MEDICAL SCIENCES FOR THE

### West of England and South Wales

PUBLISHED UNDER THE AUSPICES OF

*THE BRISTOL MEDICO-CHIRURGICAL SOCIETY*

EDITOR

J. A. NIXON, C.M.G., M.D., F.R.C.P.

WITH WHOM ARE ASSOCIATED

A. RENDLE SHORT, M.D., B.S., B.Sc., F.R.C.S.

W. MELVILLE CAPPER, M.B., B.S., F.R.C.S.

G. E. F. SUTTON, M.C., M.D., B.S., M.R.C.P.

E. WATSON-WILLIAMS, M.C., Ch.M., F.R.C.S., Editorial Secretary

---

"Scire est nescire, nisi id me

Scire alius sciret"

---

1948 *VOL. LXV*

BRISTOL: J. W. ARROWSMITH LTD.

### 1948 Contents of Vol. LXV

| | Page |
|---|---|
| Edward Jenner. Presidential Address. By T. Milling, M.B., Ch.B. .. | 1 |
| The Diagnostic Work of a Surgical Thoracic Unit, with special reference to Intra-Thoracic Suppuration and Bronchogenic Carcinoma. By R. H. R. Belsey, M.B., M.S., F.R.C.S. .. .. .. .. .. .. ... | 10 |
| A Case of Macrocheilia. By D. C. Bodenham, M.B., F.R.C.S. .. .. | 15 |
| Studies of the Renal Circulation (A Review) .. .. .. .. .. | 16 |
| Alfred, the Zoo Gorilla. By Dr. R. C. Clarke .. .. .. .. | 19 |
| Cerebral Diplegia following Birth Injury. By R. M. Norman, M.D. .. .. | 43 |
| Thyroidectomy or Thiouracil? By A. Rendle Short, M.D., B.S., B.Sc., F.R.C.S. .. .. .. .. .. .. .. .. .. .. | 48 |
| Factors in the Ætiology of Disseminated Sclerosis. By A. M. G. Campbell, D.M., M.R.C.P. .. .. .. .. .. .. .. .. .. | 54 |
| Bronchial Carcinoma. By W. W. D. Thomson, D.L., M.D., B.Sc., F.R.C.P. | 69 |
| Some Points in the Treatment of Diabetes. By G. A. Smart, M.D., B.Sc., M.R.C.P. .. .. .. .. .. .. .. .. .. .. | 76 |
| Blue Sclerotics, Fragilitas Ossium and Deafness: Report of a Family. By E. Watson-Williams, *M.C.*, M.D., Ch.M., F.R.C.S. .. .. .. | 82 |
| Thiouracil in Thyrotoxicosis .. .. .. .. .. .. .. | 87 |
| Medicine and the Law. Presidential Address (1939). By Charles Corfield, M.D., Barrister-at-Law .. .. .. .. .. | 97 |
| Tasks Ahead in Public Health. By R. H. Parry, M.D., F.R.C.P., D.P.H. .. | 102 |
| Some Experiences in the Treatment of Arterial Injuries. By J. J. Mason Brown, *O.B.E.*, M.B., Ch.B., F.R.C.S. .. .. .. .. .. | 105 |
| Clinical Note—Folic Acid .. .. .. .. .. .. .. .. .. | 111 |
| Surgical Treatment in Hypertension. By Horace Evans, F.R.C.P. .. | 112 |
| Reviews of Books .. .. .. .. .. .. .. | 21, 58, 88, 114 |
| Editorial Notes .. .. .. .. .. .. .. .. | 23, 61, 113 |
| Editor's Report .. .. .. .. .. .. .. .. .. | 24 |
| Obituaries .. .. .. .. .. .. .. .. .. | 27, 62 |
| Meetings of Societies .. .. .. .. .. .. | 28, 63, 90, 116 |
| The Medical Library of the University of Bristol .. .. | 29, 64, 93 |
| Publications Received .. .. .. .. .. .. .. | 31, 66, 95 |
| Local Medical Notes .. .. .. .. .. .. | 32, 66, 96, 117 |

### 1948 Index to Vol. LXV

Ætiology of Disseminated Sclerosis.—A. M. G. Campbell, 54

Alexander, D. A.—Obituary, 62

Alfred, the Zoo Gorilla.—Dr. R. C. Clarke, 19

Almanack.—Editorial Note, 113

Arterial Injuries, Some Experiences in the Treatment of.—J. J. Mason Brown, 105

Belsey, R. H. B.—The Diagnostic Work of a Surgical Thoracic Unit, with special reference to Intra-Thoracic Suppuration and Bronchogenic Carcinoma, 10

Birth Injury, Cerebral Diplegia following.—R. M. Norman, 43

Blue Sclerotics, Fragilitas Ossium and Deafness: Report of a Family.—E. Watson-Williams, 82

Bodenham, D. C.—A Case of Macrocheilia, 15

Books, Reviews of, 21, 58, 88, 114

Bronchial Carcinoma.—R. H. B. Belsey, 10: W. W. D. Thomson, 69

Campbell, A. M. G.—Factors in the Ætiology of Disseminated Sclerosis, 54

Carcinoma, Bronchial.—R. H. B. Belsey, 10: W. W. D. Thomson, 69

Carwardine, Thomas.—Obituary, 27

Cerebral Diplegia following Birth Injury.—R. M. Norman, 43

Clarke, Dr. R. C.—Alfred, the Zoo Gorilla, 19

Corfield, C.—Medicine and the Law, 97

Deafness, Blue Sclerotics, Fragilitas Ossium and: Report of a Family.—E. Watson-Williams, 82

Diabetes, Some Points in the Treatment of.—G. A. Smart, 76

Diagnostic Work of a Surgical Thoracic Unit, with special reference to Intra-Thoracic Suppuration and Bronchogenic Carcinoma, The.—R. H. R. Belsey, 10

Disseminated Sclerosis, Factors in the Ætiology of—A. M. G. Campbell, 54

Editorial Notes, 23, 61, 113
- Almanack, 113
- Sheriff of Bristol, 23
- Swiney Prize, 23
- Vale atque ave, 61
- The Quads, 61
- The *Journal*, 113
- Medical School Dinner, 113
- Books for Hanover, 114

Editor's Report, 24

Folic Acid—Clinical Note 110

Fragilitas Ossium and Deafness, Blue Sclerotics: Report of a Family.—E. Watson-Williams, 82

Gorilla, Alfred the Zoo, 19

Hanover, Books for.—Editorial Note, 116

Hypertension, Surgical Treatment in, 111

Jenner, Edward: Presidential Address. T. Milling, 1

*Journal*, The.—Editorial Note, 116

Law, Medicine and the.—C. Corfield, 97

Library, Medical, of the University of Bristol, 29, 64, 93

Local Medical Notes, 32, 66, 96, 117

Macrocheilia, A Case of.—D. C. Bodenham, 15

Mason Brown, J. J.—Some Experiences in the Treatment of Arterial Injuries, 105

Medical Library of the University of Bristol, 29, 64, 93

Medical Notes, Local, 32, 66, 96, 117

Medical School Dinner.—Editorial Note, 113

Medicine and the Law.—C. Corfield, 97

Meetings of Societies, 28, 63, 90, 116

Milling, T.—Edward Jenner: Presidential Address, 1

Moore, C. A.—Obituary, 27

Norman, R. M.—Cerebral Diplegia following Birth Injury, 43

### Index

Obituaries :
Thomas Carwardine, 27
C. A. Moore, 27
David Alfred Alexander, 62

Parry, R. H.—Tasks Ahead in Public Health, 102

Presidential Addresses : C. Corfield, 97; T. Milling, 1; R. H. Parry, 102

Public Health, Tasks Ahead in.—R. H. Parry, 102

Publications Received, 31, 66, 95

Quads, The.—Editorial Note, 61

Renal Circulation, Studies of the.—J. Trueta, A. E. Barclay, K. J. Franklin and M. M. L. Pritchard, 16

Report, Editor's, 24

Reviews of Books, 21, 58, 88, 113

Sclerotics, Blue, Fragilitas Ossium, and Deafness: Report of a Family.—E. Watson-Williams, 82

Sheriff of Bristol.—Editorial Note, 23

Short, A. Rendle—Thyroidectomy or Thiouracil ?, 48

Smart, G. A.—Some Points in the Treatment of Diabetes, 76

Societies, Meetings of,
Anæsthetists of South Western Region 91
Bristol Medico-Chir. 82, 63, 90
B.M.A., Bath Division, 91
Bath, Bristol and Som. Branch 116
Cardiff Medical Society, 92
Laryngological Association: South-Western, 91, 116
Medical Officers of Health, 96
Medical Records Officers, Association of, 67
Torquay and District Medical, 92
Royal Society of Medicine: Laryngology and Otology, 90
Wessex Rahere Club, 92

Some Experiences in the Treatment of Arterial Injuries.—J. J. Mason Brown, 105

Some Points in the Treatment of Diabetes.—G. A. Smart, 76

Studies of the Renal Circulation.—J. Trueta, A. E. Barclay, K. J. Franklin and M. M. L. Pritchard, 16

Subscribers.—Editorial Note, 61

Suppuration, Intra-thoracic, and Bronchogenic Carcincoma.—R. H. R. Belsey, 10

Surgical Treatment in Hypertension, 111

Swiney Prize.—Editorial Note, 23

Tasks Ahead in Public Health.—Prof. R. H. Parry, 102

The Diagnostic Work of a Surgical Thoracic Unit, with special reference to Intra-Thoracic Suppuration and Bronchogenic Carcinoma.—R. H. R. Belsey, 10

Thiouracil in Thyrotoxicosis, 87

Thomson, W. W. D.—Bronchial Carcinoma, 69

Thoracic Unit, The Diagnostic Work of a Surgical—R. H. R. Belsey, 10

Thyroidectomy or Thiouracil ?—A. Rendle Short, 48

University of Bristol, The Medical Library of the, 29, 64, 93

Vale atque ave.—Editorial Note, 61

Watson-Williams, E.—Blue Sclerotics, Fragilitas Ossium and Deafness : Report of a Family, 82

### BRISTOL MEDICO-CHIRURGICAL SOCIETY

### INCOME AND EXPENDITURE ACCOUNT for the year ended 30th September, 1947

### EXPENDITURE

| | £ | s. | d. | £ | s. | d. |
|---|---|---|---|---|---|---|
| To Subscriptions to *Medico-Chirurgical Journal* handed over (305 members at 16s. each) .. .. | | | | 244 | 0 | 0 |
| ,, Past subscriptions due to *Medico-Chirurgical Journal* (to be approved) .. .. .. .. .. | | | | 84 | 13 | 11 |
| ,, Past subscriptions due to Medical Library .. .. .. | | | | 25 | 0 | 0 |
| ,, Librarian, Honorarium .. .. | | | | 15 | 0 | 0 |
| ,, Secretary .. .. .. .. .. .. | | | | 10 | 0 | 0 |
| ,, Audit Fee .. .. .. .. .. .. | | | | 5 | 5 | 0 |
| ,, Printing and Stationery .. .. | | | | 22 | 4 | 6 |
| ,, Lewis's Medical and Scientific Library .. .. .. .. .. .. | | | | 6 | 7 | 6 |
| ,, Catering (8 times at £11 5s. a time) .. | 90 | 0 | 0 | | | |
| ,, Other Expenses of Meetings .. .. | 7 | 7 | 0 | | | |
| ,, Postages and Sundries | 23 | 9 | 0 | | | |
| | | | | 120 | 16 | 0 |
| | | | | £533 | 6 | 11 |

### INCOME

| | £ | s. | d. |
|---|---|---|---|
| By Members' Subscriptions .. .. | 325 | 10 | [illegible] |
| ,, Interest on Bank Deposit Account .. .. .. .. .. | | 4 | [illegible] |
| ,, Interest on £500 4 per cent. Consolidated Stock, less Tax .. | 11 | 0 | [illegible] |
| ,, Deficiency for the Year .. | 196 | 12 | [illegible] |
| | £533 | 6 | [illegible] |

### BALANCE SHEET as at 30th September, 1947

### LIABILITIES

| | £ | s. | d. | £ | s. | d. |
|---|---|---|---|---|---|---|
| Sundry Creditors (Auditors) .. .. .. | | | | 5 | 5 | 0 |
| Accumulated Fund as at 30th Sept., 1946 .. | 837 | 8 | 2 | | | |
| *Less* Deficiency for the year .. .. .. .. .. | 196 | 12 | 3 | | | |
| | | | | 640 | 15 | 11 |
| | | | | £646 | 0 | 11 |

### ASSETS

| | £ | s. | d. | £ | s. | d. |
|---|---|---|---|---|---|---|
| Cash at Bank— | | | | | | |
| Current Account .. | 8 | 13 | 7 | | | |
| Deposit Account .. | 90 | 14 | 4 | | | |
| | | | | 99 | 7 | [illegible] |
| £500 4 per cent. Consolidated Stock at cost .. .. .. .. .. | | | | 437 | 9 | [illegible] |
| Arrears of Subscriptions .. .. | | | | 109 | 4 | [illegible] |
| | | | | £646 | 0 | [illegible] |

Audited and found correct,

H. E. KEELER,

*Chartered Accountant.*

*Auditor.*

16–18 Clare Street, Bristol 1.

*October*, 1947.

### The Medical Library of the University of Bristol

WITH WHICH IS INCORPORATED

**The Library of the Bristol Medico-Chirurgical Society**

---

*The following donations have been received since the publication of the list in the last issue.*

| | |
|---|---|
| Association of American Physicians (1) .. .. .. | 1 volume. |
| Michell Clarke Fund (2) .. .. .. .. .. | 12 volumes. |
| Middlesex Hospital Medical School (3) .. .. .. | 1 volume. |
| Professor A. V. Neale (4) .. .. .. .. .. | 13 volumes. |
| Roche Products Ltd. (5) .. .. .. .. .. | 1 volume. |
| J. E. Shaw Fund (6) .. .. .. .. .. | 1 volume. |
| University of Texas (7) .. .. .. .. .. | 1 volume. |
| Wellcome Historical Medical Museum (8) .. .. | 2 volumes. |

Unbound periodicals have also been received from the British Medical Association, Professor T. F. Hewer, Professor R. Milnes Walker and Messrs. John Wright & Sons Ltd.

### THE ONE HUNDRED AND EIGHTY-SEVENTH LIST OF BOOKS.

The figures in round brackets refer to the figures after the names of the donors. The books to which no such figures have been attached have either been bought from the Library Fund or received through the *Journal*.

**Adams, R. C.** .. .. *Intravenous Anaesthesia* .. .. .. .. .. .. .. 1944
**Barell, E. C.** .. .. *Jubilee Volume* .. .. .. .. .. .. .. (5) 1946
**Beaumont, G. E.** .. *Medicine* .. .. .. .. .. .. .. .. 5th Ed. (2) 1948
**Berkeley, Sir C., and Bonney, V.** *Text-book of Gynaecological Surgery* 5th Ed. 1947
**Buxton, O. V., and Mackay, P. M. M.** *The Nursing of Tuberculosis* .. .. 1947
**Cameron, A. T.** .. .. *Text-book of Biochemistry* .. .. .. .. 6th Ed. 1945
**Carling, Sir E. R., and Ross, J. P.** *British Surgical Practice* .. Vol. 2 (6) 1948
**Cassidy, Sir M.** .. .. *Coronary Disease—Harveian Oration*, 1946 .. (4) 1946
*Catalogue of Medical Films*, compiled by the Royal Society of Medicine and the Scientific Film Association .. .. .. 1948
**Cecil, R. L. (Ed.)** .. *Text-book of Medicine* .. .. .. .. .. 7th Ed. 1947
**Collier, H. E.** .. .. *Outlines of Industrial Medical Practice* .. (4) 1940
**Comrie, J. D.** .. .. *History of Scottish Medicine* .. .. 2 vols. (8) 1932
**Conel, J. L.** .. .. .. *Post-natal Development of Human Cerebral Cortex* Vol. 3—*Cortex of Three-month Infant* .. (2) 1947
**Coope, R.** .. .. .. .. *Diseases of the Chest* .. .. .. .. 2nd Ed. (2) 1948
**Daley, R., and Miller, H. G. (Eds.)** *Progress in Clinical Medicine* .. (2) 1948
**De Lee, J. B., and Greenhill, J. P.** *Principles and Practice of Obstetrics* 9th Ed. (2) 1947
**Donaldson, J. K.** .. *Surgical Disorders of the Chest* .. .. 2nd Ed. 1947
**Drummond, Sir J.** .. *The Nutritive Value of Bread* (*Sanderson Wells Lecture*) .. .. .. .. .. .. .. .. .. .. (3) 1947
**Engel, S.** .. .. .. *The Child's Lung* .. .. .. .. .. .. .. .. .. 1947
**Eyre, H.** .. .. .. *An Account of the Mineral Waters of Spa* .. 1733
**Fairbairn, J. S.** .. .. *Gynaecology with Obstetrics* .. .. .. .. .. (4) 1924
**Ford, E. B.** .. .. .. *Genetics for Medical Students* .. .. 3rd Ed. (2) 1948
**French, J.** .. .. .. *The Yorkshire Spaw, or a Treatise of Four Famous Medicinal Wells* .. .. .. .. .. .. .. .. .. 1652
**Gamble, J. L.** .. .. *Chemical Anatomy, Physiology and Pathology of Extracellular Fluid* .. .. .. .. .. .. .. 1947
**Gates, R. R.** .. .. *Human Genetics* .. .. .. .. .. 2 vols. (2) 1946
**Gilmour, S.** .. .. .. *Tuberculosis in the West Indies* .. .. .. .. .. 1947
**Glasser, O.** .. .. .. *Medical Physics* .. .. .. .. .. .. .. .. .. 1947
**Goldzieher, M. A.** .. *The Adrenal Glands in Health and Disease* .. .. 1946
**Gordon, R. G.** .. .. *Abnormal Behaviour* .. .. .. .. .. .. .. (4) 1946
**Gould, Sir A. P.** .. .. *Elements of Surgical Diagnosis* .. .. 9th Ed. 1947
**Harvey, W. C., and Hill, H.** *Milk Products* .. .. .. .. .. .. 2nd Ed. 1948
**Herrick, A. D.** .. .. *New Drugs* .. .. .. .. .. .. .. .. .. .. .. 1946
**Hill, A. B.** .. .. .. *Principles of Medical Statistics* .. .. 4th Ed. 1948
**Himsworth, H. P.** .. *Lectures on the Liver and its Diseases* .. .. .. 1947
**Jackson, C., and Jackson, C. L.** *Diseases of the Nose, Throat and Ear* .. .. 1946
**Jameson, T.** .. .. .. *A Treatise on Cheltenham Waters* .. 3rd Ed. 1814
**Jenkins, W. D.** .. .. *Dermatoses Among Gas and Tar Workers* .. .. 1948
**Jones, J.** .. .. .. *The Bathes of Bathes Ayde* .. .. .. .. .. .. 1572
**Jordan, E. O.** .. .. *Food Poisoning* .. .. .. .. .. .. .. .. (4) 1917
**Katz, L. N.** .. .. .. *Electrocardiography* .. .. .. .. .. .. .. (2) 1946
**Leake, C. D.** .. .. .. *Letheon—the Cadenced Story of Anesthesia* .. (7) 1947

| | | | |
|---|---|---|---|
| **Lewis, Sir T.** | *Clinical Electrocardiography* | 6th Ed. (4) | 1937 |
| **Lickley, J. D.** | *An Introduction to Gastro-enterology* | | 1947 |
| **MacKenna, R. M. B. (Ed.)** | *Modern Trends in Dermatology* | | 1948 |
| **Martin, C. R. A.** | *Practical Food Inspection* | 2 vols. 3rd Ed. | 1947 |
| **May, H.** | *Reconstructive and Reparative Surgery* | | 1947 |
| **Miller, E. (Ed.)** | *Neuroses in War* | (4) | 1940 |
| **Miller, H. C.** | *Insomnia* | (4) | 1930 |
| **Novak, E.** | *Gynecological and Obstetrical Pathology* | 2nd Ed. (2) | 1947 |
| **Ogilvie, R. F.** | *Pathological Histology* | 2nd Ed. (4) | 1943 |
| **Pannett, C. A.** | *Surgery* | 2nd Ed. | 1947 |
| **Peter, J.** | *A Treatise of Lewisham Wells in Kent* | | 1680 |
| **Ranson, S. W.** | *The Anatomy of the Nervous System* | 8th Ed. (2) | 1947 |
| **Reynell, C.** | *A Sermon Preached before the Contributors to the Bristol Infirmary* | | 1738 |
| **Rolleston, Sir H. D., and Moncrieff, A. A. (Eds.)** | *Industrial Medicine* | (4) | 1944 |
| **Sawyer, J.** | *Notes on Medical Education* | (4) | 1889 |
| **Selye, H.** | *Text-book of Endocrinology* | | 1947 |
| **Sherrington, Sir C.** | *The Endeavours of Jean Fernel* | | 1946 |
| **Stewart, J. P.** | *Intracranial Tumours* | (4) | 1927 |
| **Stone, K.** | *Diseases of the Joints and Rheumatism* | | 1947 |
| *Treasury of Human Inheritance*, Vol. 4, Parts 3, 4, 5 | | | 1939–47 |
| **Tredgold, A. F.** | *A Text-book of Mental Deficiency* | 7th Ed. | 1947 |
| **Vaquez, H.** | *Diseases of the Heart* | (4) | 1924 |
| **Weiss, E., and English, O. S.** | *Psychosomatic Medicine* | (2) | 1947 |
| **Willis, R. A.** | *Pathology of Tumours* | | 1948 |
| **Wright, H.** | *Sex Fulfilment in Married Women* | | 1947 |

TRANSACTIONS, REPORTS, JOURNALS, ETC.

| | | |
|---|---|---|
| Annual Report of the Smithsonian Institution, 1946 | | 1947 |
| Hospitals Yearbook, 1947 | | 1947 |
| Medical Annual, 1947 | | 1947 |
| Quarterly Cumulative Index Medicus. Vol. 40. July–December, 1946 | | 1947 |
| Transactions of the Association of American Physicians. Vol. 60 | (1) | 1947 |
| Transactions of the Medical Society of London. Vol. 64 | | 1946 |
| Yearbook of Dermatology and Syphilology, 1947 | | 1947 |
| Yearbook of Eye, Ear, Nose and Throat, 1947 | | 1947 |
| Yearbook of General Medicine, 1947 | | 1947 |
| Yearbook of General Surgery, 1947 | | 1947 |
| Yearbook of Obstetrics and Gynecology, 1947 | | 1947 |
| Yearbook of Pediatrics, 1947 | | 1947 |
| Yearbook of Radiology, 1947 | | 1947 |
| Yearbook of Urology, 1947 | | 1947 |

### Publications Received

From MESSRS. JOHN WRIGHT & SONS LTD.:

- *The Nursing of Tuberculosis*. By O. V. BUXTON, S.R.N., and P. M. MACULLOCH MACKAY, S.R.M.N. Price 7s. 6d. 1947.
- *British Journal of Surgery*. Vol. 35, No. 139. January, 1948. Subscription 42s. per annum; Single numbers, 12s. 6d. 1948.
- *Dermatoses Among Gas and Tar Workers*. By WILLIAM DAVID JENKINS, J.P., B.A., M.R.C.S., L.R.C.P. Price 25s. 1948.

From MESSRS. JOHN WRIGHT & SONS LTD.:

*Psychiatry: A Short Treatise.* By WILLIAM A. O'CONNOR, L.M.S.S.A., D.P.M. Price 35s. 1948.

*Emergency Surgery.* By HAMILTON BAILEY, F.R.C.S., F.A.C.S., F.I.C.S., F.R.S.E. 6th Edition. Part I. Price 21s. 1948.

*Surgery of the Colon and Rectum.* By Sir HUGH DEVINE, M.S., and JOHN DEVINE, M.S. Price 52s. 6d. 1948.

From MESSRS. WILLIAMS AND NORGATE LTD.:

*Sex Fulfilment in Married Women.* By HELENA WRIGHT, M.B., B.S. Price 5s. 1947.

From MESSRS. H. K. LEWIS & CO. LTD.:

*Practical Food Inspection.* By C. R. A. MARTIN. Vol. 1: *Meat Inspection.* Vol. 2: *Fish, Poultry and other Foods.* 3rd Edition. Price 36s. 1947–48.

*Milk Products.* By W. CLUNIE HARVEY, M.D., D.P.H., F.R.San.I., and HARRY HILL, F.R.San.I., A.M.I.S.E., F.S.I.A. 2nd Edition. Price 30s. 1948.

From OXFORD UNIVERSITY PRESS:

*The Natural History of Disease.* By JOHN A. RYLE, M.A., M.D., F.R.C.P. 2nd Edition. Price 22s. 6d. 1948.

*The Background of Therapeutics.* By J. HAROLD BURN, M.D., F.R.S. Price 22s. 6d. 1948.

*Psychotherapy—Its Uses and Limitations.* By D. RHODES ALLISON, M.D., M.R.C.P., and R. G. GORDON, M.D., D.Sc., F.R.C.P. Price 8s. 6d. 1948.

From MESSRS. FREDERICK MULLER LTD.:

*Clinical Endocrinology and Constitutional Medicine.* By A. P. CAWADIAS, O.B.E., M.D., F.R.C.P. Price 42s. 1948.

### Local Medical Notes

### APPOINTMENT

Professor Geoffrey Hadfield, M.D., as Sir William H. Collins Professor of Human and Comparative Pathology at the Royal College of Surgeons.

### EXAMINATION RESULTS

**University of Bristol.**—Students of the University have recently passed the following examinations :—

M.B., CH.B.—*Second Examination*: A. M. Apsimon, K. B. Arney, Margaret M. Ashton, Pearl D. Barker-Wyatt, C. Barwick, W. G. Benson, A. C. Brown, June M. Butler, G. V. Catford, D. R. Coles, P. I. Draper (with Distinction in Anatomy), M. S. Dunnill, D. H. Fox, L. Harbin, B. W. Hill, G. J. Hillier, Elisabeth Hopkins, June K. Lloyd, Ruth M. Martin, G. H. Mazey, Mary Motton, I. C. Murison, P. Norris, S. J. Palmer, D. S. Paton, Ruth E. Pawlyn, Gillian F. Pinckney, A. J. Rowland, M. N. Simmonds, R. D. Stride, A. M. Sweeting, D. W. Trump, Doreen E. D. Turner, Margaret Tysoe, P. G. J. Wilcock, D. W. Wright.

D.M.R.—*Part II*: G. R. Airth, D. Livshin, E. G. Redman, N. F. Walsh.

MIDWIFE TEACHERS' CERTIFICATE.—*Part II*: Irene M. Humphries, Sarah E. D. Stuart, Grace V. Webber.

**Royal College of Surgeons.**—The Jacksonian Prize for 1947 has been awarded to N. C. Tanner, M.B., Ch.B. (Brist.), F.R.C.S.

### Bristol Medico-Chirurgical Society

### President.

T. MILLING, M.B., B.Ch. (Belf.).

### President-Elect.

H. J. DREW SMYTHE, M.C., M.D., M.S.

### Committee.

*Ex-Officio* { J. A. NIXON, *C.M.G.*, B.A., M.D. (Editor of *Journal*).

(Vacant), (Hon. Librarian).

*Elected.*

Professor MILNES WALKER.
A. PALIN, B.M., B.Ch.
H. E. HARRIS, M.B., Ch.B.
R. V. COOKE, Ch.M.
F. H. BODMAN, M.D.
G. L. ORMEROD, M.A., M.B., B.Ch.
R. F. BARBOUR, M.A., M.B., Ch.B.
LENNOX ROBERTSON, *M.C.*, M.B., Ch.B.
A. T. ROWLEY.

### Honorary Secretary.

A. L. EYRE-BROOK, M.S.

### Honorary Treasurer.

FREDERICK SUTTON, *M.C.*, M.D., F.R.C.P.

### University Library Sub-Committee.

A. R. SHORT, B.Sc., M.D.
C. BRUCE PERRY, M.D.
H. J. DREW SMYTHE, *M.C.*, M.S., M.D.

Medical Practitioners wishing to join the Society are requested to communicate with the Hon. Secretary, Mr. A. L. Eyre-Brook, Litfield House, Clifton Down, Bristol 8. The Annual Subscription is £1 11s. 6d., payable to Hon. Treasurer.

*Extracts from Journal By-laws.*

4.—The *Journal* shall be delivered free to Subscribers and also to Members of the Society whose subscriptions are not in arrear.

6.—The Annual Subscription to the *Journal* is 16/–, payable to Hon. Treasurer.

Communications intended for insertion in the *Journal* should be sent to the Editor, Dr. J. A. Nixon, 7 Lansdown Place, Clifton, Bristol.

Books for Review and Exchange Journals should be sent to the Assistant Editor, *Bristol Medico-Chirurgical Journal*, Medical Library, The University, Bristol. Reviews should be returned with the books to Mr. W. M. Capper, Litfield House, Clifton Down, Bristol 8.

Communications referring to the delivery of the *Journal* should be sent to the Hon. Treasurer, Dr. Frederick Sutton, 1 Pembroke Road, Bristol 8.

Volumes of the *Journal* can be bound upon application to J. W. Arrowsmith Ltd., Quay Street, Bristol, price on request. The inclusive Index for Volumes I—L may be obtained, price 3/– (postage extra).

### Bristol Medico-Chirurgical Society

### PAST PRESIDENTS

1874—75. FREDERICK BRITTAN, M.D., B.S. Dub.
1875—76. ROBERT WILLIAM COE.
1876—77. SAMUEL MARTYN, M.D. Ed.
1877—78. AUGUSTIN PRICHARD, M.D. Berlin.
1878—79. HENRY EDWARD FRIPP, M.D. Würzburg.
1879—80. WILLIAM MICHELL CLARKE.
1880—81. JOSEPH GRIFFITHS SWAYNE, M.D. Lond.
1881—82. EDWARD LONG FOX, M.D. Oxon.
1882—83. JAMES GEORGE DAVEY, M.D. St. And.
1883—84. WILLIAM JOHNSTONE FYFFE, M.D., B.A. Dub.
1884—85. GEORGE FORSTER BURDER, M.D. Aberd.
1885—86. WILLIAM HENRY SPENCER, M.D., M.A. Cantab.
1886—87. FRANCIS POOLE LANSDOWN.
1887—88. LEMUEL MATTHEWS GRIFFITHS.
1888—89. ROBERT SHINGLETON SMITH, M.D., B.Sc. Lond'
1889—90. NELSON CONGREVE DOBSON, Ch.M. Brist.
1890—91. SAMUEL HENRY SWAYNE.
1891—92. FRANCIS RICHARDSON CROSS, M.B. Lond., LL.D. Brist.
1892—93. EDWARD MARKHAM SKERRITT, M.D., B.S., B.A. Lond.
1893—94. JAMES GREIG SMITH, M.B., C.M., M.A. Aberd., F.R.S.E.
1894—95. ALFRED JAMES HARRISON, M.B. Lond.
1895—96. ARTHUR WILLIAM PRICHARD.
1896—97. ALFRED EDWARD AUST LAWRENCE, M.D., C.M. Aberd.
1897—98. JOHN EDWARD SHAW, M.B., C.M. Ed.
1898—99. ROBERT ROXBURGH, M.B. Ed.
1899—00. WILLIAM HENRY HARSANT.
1900—01. DAVID SAMUEL DAVIES, M.D. Lond.
1901—02. BARCLAY JOSIAH BARON, M.B., C.M. Ed.
1902—03. GEORGE MUNRO SMITH, M.D. Brist.
1903—04. JAMES PAUL BUSH, C.M.G., C.B.E., Ch.M. Brist.
1904—05. REGINALD EAGER, M.D. Lond.
1905—06. JOHN DACRE.
1906—07. JAMES TAYLOR.
1907—08. HENRY WALDO, M.D., C.M. Aberd.
1908—09. JOHN MICHELL CLARKE, M.D., M.A. Cantab.
1909—10. JAMES SWAIN, C.B., C.B.E., M.D., M.S. Lond.
1910—11. BERTRAM MITFORD HERON ROGERS, M.D., B.Ch., B.A. Oxon.
1911—12. CHARLES ALEXANDER MORTON.
1912—13. WALTER CARLESS SWAYNE, M.D., B.S. Lond. and Brist.
1913—14. PATRICK WATSON-WILLIAMS, M.D. Lond., M.D. (Hon. Causa) Brist.
1914—15. WILLIAM HARRY CHRISTOPHER NEWNHAM, M.A., M.B. Cantab
1915—16. GEORGE PARKER, M.A., M.D. Cantab., LL.D. Brist.
1916—17. FRANCIS HENRY EDGEWORTH, M.A., M.D. Cantab., D.Sc. Lond.
1917—18. FREDERICK LACE.
1918—19. ROBERT GUTHRIE POOLE LANSDOWN, M.D., B.S. Durh.
1919—20. I. WALKER HALL, M.D., Ch.B. Vict.
1920—21. LEOPOLD ERNEST VALENTINE EVERY-CLAYTON, M.D. Lond.
1921—22. CYRIL HUTCHINSON WALKER, B.A., M.B. Cantab.
1922—23. JOHN ALEXANDER NIXON, C.M.G., B.A., M.D. Cantab.
1923—24. JOHN LACY FIRTH, M.D., M.S. Lond.
1924—25. JOHN ODERY SYMES, M.D. Lond.
1925—26. THOMAS CARWARDINE, M.S. Lond.
1926—27. ARTHUR LAUNCELOT FLEMMING, M.B., Ch.B. Brist.
1927—28. WALTER KENNETH WILLS, M.A., M.B., B.Ch. Cantab.
1928—29. HENRY LAWRENCE ORMEROD, M.D., B.Ch. R.U.I.
1929—30. CHARLES FERRIER WALTERS.
1930—31. DAVID CHARLES RAYNER, Ch.M. Brist.
1931—32. JOHN ROGER CHARLES, M.A., M.D. Cantab.
1932—33. ERNEST WILLIAM HEY GROVES, M.D., M.S., D.Sc., B.Sc. Lond.
1933—34. HERBERT ELWIN HARRIS, B.A., M.B. Cantab.
1934—35. A. RENDLE SHORT, B.Sc., M.D. Lond.
1935—36. ARTHUR ERNEST ILES, O.B.E., M.B., B.S. Lond.
1936—37. RICHARD CHRISTOPHER CLARKE, O.B.E, M.B. Lond.
1937—38. CLIFFORD A. MOORE, M.B., M.S. Lond.
1938—40. LEONARD AUGUSTUS MOORE, M.B., Ch.B. Brist.
1940—41. CHARLES CORFIELD, M.D. Durh., Barr.-at-Law.
1945—46. H. H. CARLETON, M.A., M.D. Oxon.
1946—47 H. CHITTY, M.S. Lond.
1947—48. T. MILLING, M.B., B.Ch. Belf.

1948.

### LIST OF SUBSCRIBERS

TO THE

### Bristol Medico-Chirurgical Journal.

### HONORARY MEMBER OF THE SOCIETY.

| | |
|---|---|
| SWAIN, JAMES, *C.B.*, *C.B.E.*, M.D., M.S. Lond. .. .. .. | Wyndham House, Easton-in-Gordano, Som. |

### SUBSCRIBERS.

*Those marked * are Members of the Society.*

| | |
|---|---|
| *ADAMS, A. W., M.S. Lond. .. .. .. .. .. | Elton House, Clifton, Bristol 8. |
| ADAMS, J. B. .. .. .. .. .. .. .. .. .. | Kent Lodge, Eastbourne. |
| *ALDREN, J., M.B., Ch.B. .. .. .. .. .. .. | 39 Oakfield Road, Bristol 8. |
| *ALDWINCKLE, HELEN M., M.B., Ch.B. Brist. .. | 23 Rockland Road, Downend, Bristol. |
| *ALEXANDER, A. J. P., M.D. Belf. .. .. .. .. | Winsley Sanatorium, near Bath. |
| *ALEXANDER, D. A., M.B., Ch.B. Vict. .. .. | 112 Pembroke Road, Clifton, Bristol 8. |
| ALEXANDER, G. L., B.A., M.B., B.Ch. Cantab. | West African Medical Service, Laura, via Accra, Gold Coast. |
| *ALFORD, A. F., M.B., Ch.B. Brist. .. .. | Ministry of Education, Belgrave Square, London, S.W.1. |
| *APLEY, J., M.D. Lond. .. .. .. .. .. .. | Royal United Hospital, Bath. |
| *BAER, P., M.D. Milan .. .. .. .. .. .. | 10 Redland Park, Bristol 6. |
| *BAIN, W., M.D. Glasg. .. .. .. .. .. .. | 1 Greville Road, Southville, Bristol 3. |
| *BAKER, LILY A., M.D., B.Ch., B.A.O., R.U.I. | 23 Pembroke Road, Clifton, Bristol 8. |
| BALL, Mrs. J. J. .. .. .. .. .. .. .. | St. Leonard's Vicarage, Redfield, Bristol 5. |
| *BALLANTYNE, J. C. .. .. .. .. .. .. .. | 128 Badminton Road, Downend, Bristol. |
| *BARBOUR, R. F., M.A. Camb., M.B., Ch.B. Edin. | Hill House, Iron Acton, Glos. |
| *BARNFIELD, R. H. .. .. .. .. .. | 15 The Paragon, Clifton, Bristol 8. |
| *BASTOW, J., M.D., B.S. Melb. .. .. .. .. .. | 5 The Circus, Bath. |
| *BAXTER, URSULA, M.B., Ch.B. Brist. .. .. | 334 Canford Lane, Westbury, Bristol 9. |
| *BAXTER, V. T., M.B., Ch.B. Brist. .. .. .. | 334 Canford Lane, Westbury, Bristol 9. |
| *BEAMISH, P. H. .. .. .. .. .. .. .. .. | Bristol General Hospital. |
| *BEATSON, D. H., M.B., Ch.B. Brist. .. .. | 8 Richmond Park Road, Clifton, Bristol 8 |
| *BELL, R. J. IRVING .. .. .. .. .. .. .. | 5a Oakfield Road, Clifton, Bristol 8. |
| *BELSEY, R., M.S. Lond. .. .. .. .. .. | Frenchay Hospital, near Bristol. |
| *BERGIN, F. G. .. .. .. .. .. .. .. .. | 31 Oakfield Road, Clifton, Bristol 8. |
| *BERGIN, K., M.A., M.D. Cantab. .. .. .. | 31 Oakfield Road, Clifton, Bristol 8. |
| *BERRY, R. J. A., Prof., M.D., C.M. Edin. .. | 3c All Saints' Road, Clifton, Bristol 8. |
| *BIDEN-STEELE, K., M.D. Lond. .. .. .. | 30 Pembroke Road, Bristol 8. |
| *BIRRELL, J. A., M.D., B.S. Lond. .. .. .. | 1 Victoria Square, Bristol 8. |
| *BISHOP, G. W. R., *O.B.E.*, M.B., Ch.B. Brist. | Bloomfield House, High Street, Shepton Mallet. |
| *BLACK, W. R., M.B., Ch.B. Edin. .. .. .. | Southside, Rockleaze, Bristol 9. |
| *BODENHAM, D. C., M.B., Ch.B. Brist. .. .. | 22 Royal York Crescent, Clifton, Bristol 8. |
| *BODMAN, F. H., M.D., Ch.B. Brist. .. .. .. | 2 Redland Court Road, Bristol 6. |
| *BOLT, D., M.B., Ch.B. Brist. .. .. .. .. | 33 Knowle Road, Bristol 4. |
| BOLT, R. F. .. .. .. .. .. .. .. .. .. | 4 Gordon Mansions, Torrington Place, London, W.C.1. |
| *BOULTON, B. J., M.B., Ch.B. Brist. .. .. .. | 23 Oakwood Road, Henleaze, Bristol. |

| | |
|---|---|
| *Bowles, Elizabeth M., M.B., B.S. Lond. | 4 Alma Road, Clifton, Bristol 8. |
| *Bown, Naomi J., M.B., Ch.B. Brist. | The Corner, Nailsea, Somerset. |
| *Boyd, R. G., M.B., Ch.B. Brist. | 44 Elm Lane, Bristol 6. |
| *Bradbeer, E. G., M.B., Ch.B. Brist. | 7 Avon Grove, Sneyd Park, Bristol 9. |
| *Brasher, C. W. J., M.D. Brist. | 17 Royal York Crescent, Clifton, Bristol 8. |
| *Brennen, R. G., M.B., B.Ch., B.A.O. Belf. | 19 Beaufort Road, Bristol 8. |
| *Brinckman, Winifred P., M.B., Ch.B. Brist. | 2 Clifton Park, Bristol 8. |
| *Britton, R. B., M.B., B.S. Lond. | 19 Whiteladies Road, Clifton, Bristol 8. |
| *Brocklehurst, R. J., M.A., D.M. Oxon. | The University, Bristol 8. |
| Brown, J. E., M.B., Ch.B. Brist. | Coulthurst Villa, Brives Road, Maraval, Trinidad, B.W.I. |
| *Brueton, N. F. W., M.B., Ch.B. Brist. | 40 Salisbury Road, Bristol 6. |
| Bryan, C. | 44 Nevil Road, Bishopston, Bristol 7. |
| *Bryant, Eunice | 11 Wootton Park, Knowle, Bristol 4. |
| *Bulleid, J. H. | G. E. C. Building, Kingsway, Cardiff. |
| *Burges, R. | Druid's Mead, Stoke Bishop, Bristol 9. |
| *Bush, G. B., M.B., Ch.B. Brist. | Litfield House, Clifton, Bristol 8. |
| | |
| *Cairns, F. J. | Devon House, Whitehall Road, Bristol 5. |
| *Cameron, D., M.B., Ch.B. Glasg. | 1 Dean Lane, Bristol 3. |
| Cameron, Margaret, M.B., Ch.B. Glasg. | 1 Dean Lane, Bristol 3. |
| *Cameron, W., M.B., Ch.B. Glasg. | 1 Dean Lane, Bristol 3. |
| *Campbell, A. M. G., D.M. Oxf. | 62 Pembroke Road, Clifton, Bristol 8. |
| *Capper, W. M., M.B., B.S. Lond. | 28 Downs Park West, Bristol 6. |
| *Carayanopoulos, T. N. | Bristol General Hospital. |
| *Carey, R. S., *O.B.E.*, B.A. Cantab. | 502 Bath Road, Brislington, Bristol 4. |
| *Carleton, H. H., M.A., D.M., B.Ch. Oxon. | 1 Lansdown Road, Clifton, Bristol 8. |
| *Carmichael, T. G. | 140 Wick Road, Bristol 4. |
| *Casson, A. H. | Hill House, Chipping Sodbury, Glos. |
| *Casson, E., M.D., Ch.B. Brist. | Mount Pleasant, Clevedon. |
| *Cates, R. N., M.B., B.S. Lond. | 68 Park Row, Bristol. |
| *Chambers, E. R. | 9 Clifton Park, Clifton, Bristol 8. |
| *Chapman, W. A., M.B., Ch.B. | 41 Salisbury Road, Redland, Bristol 6. |
| *Charles, J. R., M.A., M.D. Cantab. | Churchill Court, near Bristol. |
| *Chilcott, J. C. K., B.M., B.Ch. Oxf. | 37 Deanery Road, Warmley, near Bristol. |
| *Chitty, H., M.S. Lond. | Naish House, Clapton-in-Gordano, Som. |
| *Christofferson, P. E., M.B., Ch.B. Brist. | 14 Oakfield Road, Clifton, Bristol 8. |
| *Claremont, L. E., M.D.S. Brist. | 5 Rodney Place, Clifton, Bristol 8. |
| *Clark, D. O., M.B., B.S. Lond. | 168 Milton Road, Weston-super-Mare. |
| *Clarke, R. C., *O.B.E.*, M.B., Ch.B. Brist. | Harley Lodge, Clifton Park, Bristol 8. |
| *Clifton, F., M.B., B.Ch. Cantab. | 28 Victoria Square, Clifton, Bristol 8. |
| *Cochrane, J. A. | 11 Bryant's Hill, St. George, Bristol 5. |
| *Cole, W. R. | 5 Sea Mills Lane, Bristol 9. |
| *Collingwood, F. C., M.B., Ch.B. Brist. | 106 Foxley Lane, Purley, Surrey. |
| *Cook, Elizabeth M., M.D., Lond. | 15 Queen's Court, Clifton, Bristol 8. |
| *Cooke, R. V., Ch.M. Brist. | Litfield House, Bristol 8. |
| *Cookson, R. G. | Clifton College, Bristol 8. |
| Coombs, N. C. | 55 Galgate, Barnard Castle, Co. Durham. |
| *Cooper, W. D. G., M.B., Ch.B. | Groves End House, Thornbury, Glos. |
| *Corfield, C., M.D. Durh. | 5 Kensington Place, Clifton, Bristol 8. |
| *Cormack, J. M., M.B., Ch.B. Glasg. | 1 Rodway Hill, Mangotsfield, Bristol. |
| *Corner, Beryl, M.D., B.S. Lond. | 3 Elton House, Rodney Place, Bristol 8. |
| *Cossens, K. M., B.Chir. Camb. | 52 Cotham Hill, Cotham, Bristol 6. |
| *Cossham, D. G., M.B., Ch.B. Brist. | Kingsleigh, Cirencester. |
| *Cossham, W. L., M.B., Ch.B. Brist. | 3 Claremont Road, Bishopston, Bristol 7. |
| *Courtney, R. M., M.B., Ch.B. Glasg. | Manor House, Southmead Road, Bristol 9. |
| *Coxon, Beatrice | 16 Richmond Hill, Clifton, Bristol 8. |
| *Craig, Anne A., M.D., B.S. Lond. | 35 Queen's Court, Clifton, Bristol 8. |
| *Craig, N. Smith, M.B., Ch.B. Edin. | 7 Miles Road, Clifton, Bristol 8. |
| *Crook, B. A., M.B., Ch.B. Brist. | The Laurels, Timsbury, near Bath. |
| *Croot, H. J., M.B., B.S. Lond. | Dept. of Surgery, Royal Infirmary, Bristol 2 |
| *Crossman, F. W., M.B. Durh. | White's Hill, Hambrook, near Bristol. |
| *Curwen, S. | Bristol General Hospital. |

| | |
|---|---|
| Dammrel, Mrs. M.B., Ch.B. .. .. .. .. | Henley Lodge, Yatton, Som. |
| *Darling, Prof. A. I., M.D.S. .. .. .. .. | Bristol Dental Hospital, Bristol 1. |
| *Datta, K., M.B., Ch.B. Brist. .. .. .. .. | Snowdon House, Fishponds, Bristol. |
| *Datta, S. S., M.D. Brist. .. .. .. .. .. | Snowdon House, Fishponds, Bristol. |
| *Davies, D. H., M.B. .. .. .. .. .. .. .. | Charlton, Leigh Woods, Bristol. |
| *Davies, E. D. D. .. .. .. .. .. .. .. | Battledown Court, Cheltenham, Glos. |
| *Davies, J. N. P., M.B., Ch.B. Brist. .. .. | Path. Department, Mulago Hospital, P.O. 351, Kampala, Uganda. |
| *Davy, B. W., M.D. Lond. .. .. .. .. .. | Kingsheath, Westbury Hill, Westbury, Bristol 9. |
| *Davy, D. M., M.B., Ch.B. Brist. .. .. .. | Kingsheath, Westbury Hill, Westbury, Bristol 9. |
| *Delaney, N., M.B., Ch.B. Manch. .. .. .. | The Meade, Bishopsworth, Somerset. |
| *Dickson, R. R., M.B., B.S. Lond. .. .. .. | 50 Ridgeway Road, Long Ashton, Bristol. |
| *Disney, M., M.D. Lond. .. .. .. .. .. | |
| *Dix, C. .. .. .. .. .. .. .. .. .. .. | 14 Mortimer Road, Clifton, Bristol 8 |
| *Dixon, Mary, M.B., Ch.B. Brist. .. .. .. | 11 Pembroke Road, Bristol 8. |
| *Dixon, T. B., *C.B.E.*, V.D. .. .. .. .. | 11 Pembroke Road, Bristol 8. |
| *Dodd, G., M.B., B.S. Durh. .. .. .. .. | c/o Llanelly General Hospital, Marble Hall Road, Llanelly, Carm. |
| *Dowling, A.A., M.B., Ch.B. Brist. .. .. .. | 13 Dowry Square, Bristol 8. |
| *Duerden, J. R., M.B., Ch.B. Brist. .. .. | 4 Cecil Road, Bristol 8. |
| *Dunster, Marjorie, M.D. Lond., Ch.M. Brist. | Bristol General Hospital (St. Monica's). |
| *Dutton, Hilda R. .. .. .. .. .. .. .. | 405 Stapleton Road, Bristol 5. |
| | |
| *Eastes, H. J., M.B., B.S. Lond. .. .. .. | Bank House, Marshfield, Glos. |
| *Eastes, Zeta .. .. .. .. .. .. .. .. | Bank House, Marshfield, Glos. |
| *Eglinton, J. H. C. .. .. .. .. .. .. .. | Street, Somerset. |
| *Elliott, Lt. Com. John, M.B., Ch.B. Brist. .. | Southmead Hospital, Bristol. |
| *Elliott, Sq.-Leader W. H. Greville, M.B., Ch.B. Brist. .. .. .. .. .. .. .. .. | 100 Redland Road, Bristol 6. |
| *Ellis, W. T. .. .. .. .. .. .. .. .. | Tregenna House, Shirehampton, Bristol. |
| *Emery, J. L., M.D. Brist. .. .. .. .. .. | Children's Hospital, Sheffield. |
| *Evans, C., *O.B.E.*, M.B., B.Ch. Camtab. .. | Litfield House, Clifton Down, Bristol 8. |
| *Evans, G. M., M.B., Ch.B. Brist. .. .. .. | 117 Ashley Road, Bristol. |
| *Eyre-Brook, A. L., M.B., M.S. Lond. .. .. | Litfield House, Bristol 8. |
| *Eyre-Brook, E. M., M.B., Ch.B. Brist. .. .. | 21 Druid Road, Stoke Bishop, Bristol 9. |
| | |
| *Fairman, H. D., M.B., B.S. Lond. .. .. .. | 50 Reedley Road, Bristol 9. |
| *Fairweather, D. S., M.A., M.D. Edin. .. .. | Purdown House, Stapleton, Bristol. |
| *Fayle, B. W. D., B.A., M.B., B.Ch., B.A.O. Dub. | Galen House, Dursley, Glos. |
| *Fells, G. R., M.B., Ch.B. Brist. .. .. .. | 19 Southmead Road, Bristol 9. |
| *Fells, R. R., M.B., B.S. Lond. .. .. .. | 17 Mortimer Road, Clifton, Bristol 8. |
| *Feneley, G. L., M.B., Ch.B. Brist. .. .. | Avon Lodge, 8 Parry's Lane, Bristol 9. |
| *Field, J. G., M.B., Ch.B. Brist. .. .. .. | Buckingham Lodge, Keynsham, near Bristol. |
| *Finzel, H. F. M., M.D. Brist. .. .. .. .. | The Beeches, St. Anne's Park, Bristol 4. |
| *FitzGibbon, G. M., M.D. Lond. .. .. .. | 1 Stoke Hill, Bristol 9. |
| *Flemming, A. A. G., *O.B.E.* .. .. .. .. | Bristol General Hospital, Bristol. |
| *Forrester-Brown, Maud, M.D., M.S. Lond. | 22 Combe Park, Bath. |
| *Foss, G. L., M.D., B.Ch. Cantab. .. .. .. | 1 Cambridge Park, Bristol 6. |
| *Foss, Miss Joan, M.B., Ch.B. Brist. .. .. | Clouds Hill House, St. George, Bristol 5. |
| *Fraser, A. D., M.A., M.B., Ch.B. Glasg. .. | 6 Rayleigh Road, Coombe Dingle, Bristol. |
| *Fraser, C. Jean, M.B., Ch.B. Bristol .. .. | 6 Rayleigh Road, Coombe Dingle, Bristol. |
| *Fraser, D., M.B., Ch.B., Edin. .. .. .. .. | Westgate, Dursley, Glos. |
| *Fuller, H. L. .. .. .. .. .. .. .. .. | 9 Gay Street, Bath. |
| | |
| *Garden, R. R., M.A., M.B., B.Ch. Aberd. .. | Camp House, Clifton Down, Bristol 8. |
| Garland, E. B., M.B., C.M. Edin. .. .. .. | 114a Hampton Road, Redland, Bristol 6. |
| *Gaskell, K. H. .. .. .. .. .. .. .. .. | The Old House, Frenchay, near Bristol. |
| *Gerrish, N., M.B., Ch.B. Brist. .. .. .. | 2 Station Road, Keynsham, Som. |
| *Gibbs, J. R., M.B., Ch,B. Brist. .. .. .. | |

| | |
|---|---|
| *Gibson, B. F., M.B., B.S. Durh. | Nare House, Birchwood Road, Bristol 4. |
| *Glaser, S., M.Sc. Cape Town, M.B.B.S., Lond. | Sunnyside, Weston Road, Bath. |
| *Golding, H. M., *D.F.C.*, M.B., Ch.B. Brist. | 10 Barton Hill Road, Bristol 5. |
| *Gordon, R. G., M.D. Edin. | 23 Queen's Square, Bath. |
| *Gorham, A. P., M.B., Ch.B. Brist. | 53 Westbury Road, Westbury, Bristol 9. |
| *Gornall, W. A., M.D. Brist. | Ulvik, Nailsea, near Bristol. |
| *Graham, J. M., M.B., Ch.B. | 1 Manor Road, Fishponds, Bristol. |
| *Gray, D. R. | Wrington, near Bristol. |
| Griffiths, C. A. | 35 Newport Road, Cardiff. |
| | |
| *Hadden, R., C.M. | Royal Devon and Exeter Hospital, Exeter. |
| *Ham, C. I., M.B., Ch.B. Brist. | 89 Stonebridge Park, Eastville, Bristol. |
| *Harris, H. E., M.A., M.B., B.Ch. Cantab. | 13 Lansdown Place, Clifton, Bristol 8. |
| *Hartley, Greta, M.D. Lond. | 19 Queen's Court, Clifton, Bristol 8. |
| *Hawkins, Monica, M.B., Ch.B. Brist. | 7 Queen's Court, Bristol 8. |
| Heales, J. N., M.B., Ch.B. Brist. | 27 Clevedon Road, Weston-super-Mare. |
| *Hector, F., M.D. Lond. | 1 Victoria Square, Clifton, Bristol 8. |
| *Hemphill, R., M.D. Dub. | Clonora, Blackberry Hill, Fishponds. |
| *Henderson, J., M.B., Ch.B. Aberd. | The City Sanatorium, Yardley Road, Birmingham. |
| *Heron, A. G., M.B., Ch.B. | 237 Gloucester Road, Bristol 7. |
| *Hewer, T. F., M.D. Brist. | Canynge Hall, Bristol 8. |
| *Hickey, M. P. | 3 Mortimer Road, Clifton, Bristol 8. |
| *Hill, L. C., M.D. Birm. | 11 The Circus, Bath. |
| *Hiscock, Winifred | Broken Hill, Abbots Leigh, near Bristol. |
| *Hobson, W., M.D. Leeds | Rock Cottage, Marlborough Hill, Bristol 2. |
| *Hoffman, H. L., B.A., M.D., B.Ch. Cantab. | 26 The Circus, Bath. |
| *Holland, L. C. | Upavon, Wilts. |
| *Holland, W. A. L. | 1 Upper Belgrave Road, Clifton, Bristol 8. |
| *Hooker, J. A., Col., M.B., Ch.B. Brist. | Northlands, Cowes, I.O.W. |
| *Hosken, J. G. F. | Uley, Glos. |
| Hospital, Bristol General | |
| *Houghton, Lydia M., M.B., B.S. Lond. | 74 Church Road, Redfield, Bristol 5. |
| *Howell, T. E. | 6 Richmond Hill, Bristol 8. |
| *Hudson, T. G. F., M.D. Lond. | 103 Pembroke Road, Clifton, Bristol 8. |
| *Hughes, F. W. T., M.B., Ch.B. Brist. | Chew Magna, Somerset. |
| *Hunter, F. S. | Penrose Cottage, Clifton Down, Bristol 8. |
| | |
| *Iles, Anna, M.B., Ch.B. Brist. | 87 Pembroke Road, Clifton, Bristol 8. |
| *Iles, A. E., *O.B.E.*, M.B., B.S. Lond. | 87 Pembroke Road, Clifton, Bristol 8. |
| Infirmary, Bristol Royal | |
| Ingle, C. D. | Somerton, Somerset. |
| *Isserlin, B., M.B., Ch.B. Bristol | 21 St. John's Road, Bristol 8. |
| | |
| *Jackman, W. A., *T.D.*, M.B., Ch.B. Brist. | 15 Mortimer Road, Clifton, Bristol 8. |
| *James, J. A., M.D. Lond. | Litfield House, Clifton Down, Bristol 8. |
| *Jenkins, F. G., M.B., Ch.B. Brist. | 51 Redcliff Hill, Bristol 1. |
| *Jenkins, P. K., *M.C.*, M.B., Ch.B. Brist. | St. Helena, Marine Parade, Clevedon. |
| *Johnson-Mason, J. | Brentry House, Westbury, Bristol. |
| *Johnson, R. G., M.B. Lond. | 119 Redland Road, Bristol 6. |
| *Jones, D. M., M.Ch. | Sutton House, Clifton Down, Bristol 8. |
| *Jones, Megan E. | Royal Infirmary, Bristol 2. |
| *Joscelyne, P. C., M.B., Ch.B. Brist. | 17 Falcondale Road, Westbury, Bristol 9. |
| | |
| *Kempton, J. J., M.D. Brist. | Tudor House, Wokingham, Berks. |
| *Kersley, G. D., M.D. Cantab. | 6 The Circus, Bath. |
| *Kindersley, C. E., M.B., Ch.B., B.A. Cantab. | 18 The Circus, Bath. |
| King, E. F. | 79 Harley Street, London, W.1. |
| *Kingston, S. H., M.B., Ch.B. Brist. | 3 Whiteladies Road, Clifton, Bristol 8. |
| *Komocki, F/Lt. C., M.B. | 39 Headley Park Avenue, Bristol 3. |
| *Kyle, H. G., M.A., M.D., B.Ch. Oxon. | 31 Westbury Road, Bristol. |

*LACE, E. J. .. .. .. .. .. .. .. .. 15 Julian Road, Bristol 9.
*LACE, F. .. .. .. .. .. .. .. .. 5 Gay Street, Bath.
*LACE, M. E., M.B., Ch.B. Brist. .. .. .. 15 Julian Road, Bristol 9.
*LEES, E. L., M.D., C.M. Edin. .. .. .. 28 Downs Park West, Bristol 6.
*LEGAT, P., M.B., Ch.B. Brist. .. .. .. .. Cossham Memorial Hospital, Kingswood, Bristol.
*LEITCH, LOIS M., M.B., Ch.B. Brist. .. .. 242 Henleaze Road, Bristol.
*LENTON, P. H., M.A., M.B., B.Chir. Cantab. Royal Infirmary, Bristol.
*LEWIS, F. J., M.B., Ch.B. Brist. .. .. .. .. Canynge Hall, Bristol 8.
*LEWIS, J. K., M.B., Ch.B. Brist. .. .. .. .. 16 Elmdale Road, Clifton, Bristol 8.
*LEWIS, WINIFRED, M.B., Ch.B. Brist. .. .. Brooklands, Keesdale Road, Leytonstone, London, E.11.
*LOXTON, S. D., M.B., Ch.B. Brist. .. .. .. .. City Hospital, Nottingham.
*LUCAS, J. E. .. .. .. .. .. .. .. .. .. c/o 132 York Road, Bristol 3.
*LUCAS, J. J. S., M.D. Lond. .. .. .. .. 186 Wells Road, Knowle, Bristol 4.
*LUCAS, J. V., M.A., M.B., B.Ch. Cantab. .. 186 Wells Road, Knowle, Bristol 4.
*LUCAS, R. P., M.B., Ch.B. Brist. .. .. .. Northways, Fallodon Way, Bristol 9.

*MCKENZIE, W. G., *M.C.* .. .. .. .. .. .. 11 Bath Road, Reading.
*MACLACHLAN, A. M., M.B., Ch.B. Glasg. .. 65 Redcliff Hill, Bristol 1.
*MACLAREN, CATHERINE J., M.B., Ch.B. Edin. Hereford House, Clifton Down, Bristol 8.
MACLEAN, Sir EWEN J., M.D., C.M. Edin. .. 12 Park Place, Cardiff
*MACLEOD, G., M.A. Glas., M.D., .. .. .. .. Wargrave, Clevedon, Somerset.
*MARSHALL, A. H., M.B., Ch.B. Brist. .. .. 281 Canford Lane, Bristol 9.
*MARTIN, P. S., *M.C.* .. .. .. .. .. .. .. Victoria Quadrant, Weston-super-Mare.
*MARWOOD, S. F., M.D., B.S. Lond. .. .. Troy House, Kingswood, Bristol.
*MASON, J., M.B., Ch.B. Edin. .. .. .. .. 7 Westbury Road, Westbury, Bristol 9.
*MAWSON, EVELYN, M.D. Liverp. .. .. .. .. Frenchay Park Sanatorium, Bristol.
*MCGOWAN, G. K., B.A., B.M., B.Ch. Oxf. .. 15 Coniston Avenue, Combe Dingle, Bristol 9.
MIALL, C. L'O .. .. .. .. .. .. .. .. .. Elm Hayes, Paulton, near Bristol.
*MILLER, A. .. .. .. .. .. .. .. .. .. Bristol Royal Infirmary.
*MILLING, T., M.B., B.Ch. Belf. .. .. .. .. 1 Vicarage Road, Southville, Bristol 3.
*MOHAN, S. L., M.B., B.S. Punjab. .. .. .. 255 Stapleton Road, Bristol 5.
*MOORE, H. D. .. .. .. .. .. .. .. .. .. Bristol Royal Infirmary.
*MOORE, R. H., M.B., Ch.B. Brist. .. .. .. .. White Cross Court, Wells Road, Bristol 4.
*MORGANS, C. C., M.A., M.B., B.Ch. Cantab. .. 65 Lower Redland Road, Bristol 6.
*MUNDY, G. S., M.B., B.Ch. Brist. .. .. .. The Knoll, Chipping Sodbury, Glos.
*MURPHY, Col. D. F., *M.C.*, M.B., N.U.I., I.M.S. (Retd.) .. .. .. .. .. .. .. .. .. 25 St. Martin's Road, Bristol 4.
*MURPHY, F. D., *O.B.E.*, B.Sc., M.B., B.Ch., B.A.O., N.U.I. .. .. .. .. .. .. .. 20 Gay Street, Bath.
*MYLES, Q. St. L., M.A., B.M., B.Ch. Oxon. .. 7 Apsley Road, Clifton, Bristol 8.

*NEALE, Prof. A. V., M.D. Birm. .. .. .. .. "Firwood," Long Ashton, Bristol.
*NETHERCOTT, A. S., M.B., Ch.B. Brist. .. .. 95 Effingham Road, St. Andrew's, Bristol 6.
*NICHOLSON-LAILEY, J. R. .. .. .. .. .. .. Elmfield House, South Road, Taunton.
*NICHOLSON, M. A., M.B., Ch.B. Brist. .. .. 2 Elmlea Avenue, Stoke Bishop, Bristol 9.
*NIXON, DOREEN, M.B., B.S. Lond. .. .. .. 7 Lansdown Place, Clifton, Bristol 8.
*NIXON, J. A., *C.M.G.*, M.D. Cantab. .. .. 7 Lansdown Place, Clifton, Bristol 8.
*NOBLE, J. I., M.B., Ch.B. Liverp. .. .. .. 20 Richmond Hill, Clifton, Bristol 8.
*NORMAN, R. M., M.D. Brist. .. .. .. .. Highwood, Stapleton Park, Bristol.
*NORRIS, C. T. .. .. .. .. .. .. .. .. 11 Bryant's Hill, Bristol 5.

*OAKES, JEAN, M.B., Ch.B. Brist. .. .. .. .. Linden Lodge, Clevedon.
*OAKES, H., M.B., Ch.B. Brist. .. .. .. .. Linden Lodge, Clevedon.
*O'CONNOR, M., M.B., B.Ch. .. .. .. .. .. Addenbrooke's Hospital, Cambridge.
*OLIFF, D. E., M.B., Ch.B. .. .. .. .. .. .. 8 Lansdown Crescent, Bath.
*ORMEROD, G. L., M.A., M.B., B.Ch. Cantab. .. 2 Henleaze Road, Westbury, Bristol 9.
*ORR-EWING, H. J., *M.C.*, M.D. Lond. .. .. 1 Beaufort Buildings, Clifton, Bristol 8.

| | |
|---|---|
| *ORTON, B. F., M.B., Ch.B. Brist. .. .. .. | 121 High Street, Hanham, Bristol. |
| *ORTON, R. W., M.B., Ch.B. Brist. .. .. .. | 121 High Street, Hanham, nr. Bristol. |
| *OWEN, R. H., M.B., Ch.B. .. .. .. .. | 51 Park Grove, Henleaze, Bristol. |
| *PAGE, K. .. .. .. .. .. .. .. .. .. .. | Sunnyhill Park Hill, Shirehampton, Bristol. |
| *PALIN, A. G., B.M., B.Ch. Oxon. .. .. .. | Litfield House, Clifton Down, Bristol 8. |
| *PARKES, P. W. J. .. .. .. .. .. .. .. | 164 Filton Avenue, Bristol 7. |
| *PARRY, R. H., M.B., B.S.Lond. .. .. .. | Public Health Offices, Bristol 1. |
| PARSONS, Sir J. H., M.B., B.S., B.Sc. Lond... | 54 Queen Anne Street, London, W. |
| *PAUL, R. G. .. .. .. .. .. .. .. .. .. | 58 St. John's Road, Bristol 8. |
| *PAULI, C. H. .. .. .. .. .. .. .. .. | Pagan's Hill Farm, Chew Stoke, Som. |
| *PAULI, M. A., M.B., B.Ch., N.U.I. .. .. .. | Pagan's Hill Farm, Chew Stoke. |
| *PEACH, A. N. H., M.B., Ch.B. Brist. .. .. | Bristol Royal Infirmary. |
| *PEARSE, H., M.D. Brist. .. .. .. .. .. .. | Southfield, Taunton. |
| *PEARSON, G. M. .. .. .. .. .. .. .. .. | West Harptree, near Bristol. |
| *PENROSE, J. H., M.B., B.Chir. Cantab. .. .. | 81 Reedley Road, Westbury, Bristol. |
| *PERKS, RUTH, M.B., Ch.B. Brist. .. .. .. | 14 The Crescent, Henleaze, Bristol. |
| *PERRY, C. B., M.D., Ch.B. Brist. .. .. .. | Royal Infirmary, Bristol. |
| *PETTY, W. J., M.B., B.S. Lond. .. .. .. | "Craigside," Worle, Weston-super-Mare. |
| PHELPS, P. C., M.B., Ch.B. Brist. .. .. .. | 23 St. Alban's Road, Bristol 6. |
| *PHILLIPS, P., M.B., Ch.B. Brist. .. .. .. | Southmead Hospital, Bristol. |
| *PIRRIE, A. L., M.B., Ch.B. Glasg. .. .. | 528 Kensington Hill, Bristol. |
| *POCOCK, J. A., M.A., M.B., B.Ch. Cantab. .. | Medical Postgraduate Dept., The University, Bristol. |
| *POND, Mrs. M. H., M.B., B.Ch. Cantab. .. | 24 Cherrington Road, Henleaze, Bristol 7 |
| *PORTER, CHARLES, M.A. Oxon. .. .. .. .. | 124a Redland Road, Bristol 6. |
| PORTER, C. P. .. .. .. .. .. .. .. .. .. | 27 Church Street, Kidderminster. |
| *POTTER, MABEL F., M.D., Brist. .. .. .. | 19 Royal Park, Clifton, Bristol 8. |
| *POWELL, H. F., M.D. Brux. .. .. .. .. .. | Ellenborough Park, Weston-super-Mare. |
| *PRATT, W. W. .. .. .. .. .. .. .. .. .. | Feltham Farm, Pucklechurch. |
| *PRICE, C. H. G., M.B., Ch.B. Brist. .. .. .. | 6 Lansdown Place, Clifton, Bristol 8. |
| *PRIDIE, K. H., M.B., B.S. Lond. .. .. .. | 58 St. John's Road, Clifton, Bristol 8. |
| *PRIDIE, Mrs. KENNETH .. .. .. .. .. .. | 58 St. John's Road, Bristol 8. |
| *PROWSE, D. C., M.B., Ch.B. .. .. .. .. .. | Wigmore House, Thornbury, Near Bristol. |
| *PYKE, H. D., M.B., Ch.B. Brist. .. .. .. | 88 Redland Road, Bristol 6. |
| | |
| *RABAN, J. P. .. .. .. .. .. .. .. .. .. | Bristol General Hospital. |
| *RANKIN, P. C., M.B., Ch.B. Glasg. .. .. .. | Lawrence Hill House, Bristol. |
| *REID, A. C., B.Sc., M.B., Ch.B. Edin. .. .. | Olveston, near Bristol. |
| *RICHARDS, F. B., M.B., Ch.B. Brist. .. .. | 23 Grange Park, Westbury, Bristol. |
| *RIDGWAY, J., M.B., Ch.B. Brist. .. .. .. | 18 Blenheim Road, Durdham Down, Bristol 6. |
| *ROBERTS, J. A. F., M.A. Cantab., M.B., Ch.B., D.Sc. Edin. | Stoke Park Colony, Bristol. |
| *ROBERTS, J. A. L., *M.C.*, Ch.B. Brist. .. .. | 75 High Street, Kingswood, Bristol. |
| *ROBERTSON, D., M.B., Ch.B. Edin. .. .. .. | Echo Gate, Saltford, near Bristol. |
| *ROBERTSON, L. G., *M.C.*, M.B., Ch.B. Brist. .. | 162 Redland Road, Bristol 6. |
| *ROGERS, H., M.D. Brist. .. .. .. .. .. .. | "Marguerite," Catlin's Lane, Eastcote, Middlesex. |
| *ROLFE, R., B.A., M.B., B.C. Cantab. .. .. | Park House, St. Andrew's Road, Avonmouth. |
| *ROSS, A. I., M.D. .. .. .. .. .. .. .. | 18 Grange Park, Westbury, Bristol. |
| *ROWE, A. R., M.B., Ch.B. Brist. .. .. .. | 81 Park Grove, Henleaze, Bristol. |
| *ROWLEY, A. T. .. .. .. .. .. .. .. .. .. | 13 Walliscote Road, Weston-super-Mare. |
| *RUDOLF, G. DE M. .. .. .. .. .. .. .. .. | Litfield House, Clifton, Bristol 8 |
| *RUSHTON, E. P. K., M.B., Ch.B., Birm. .. .. | "Turville," Pilning, Glos. |
| | |
| *SAMPSON, O. E. L., M.B., Ch.B. Brist. .. .. | 77 Stackpool Road, Bristol 3. |
| *SANSOM, J. R. E., B.Sc., M.B., Ch.B. Brist. .. | 149 Bell Hill Road, St. George, Bristol 5. |
| *SCARFF, G., M.B., Ch.B. Edin. .. .. .. .. | 83 Pembroke Road, Clifton, Bristol 8. |
| *SHEPHERD, H. L., M.B., Ch.M. Brist. .. .. | 79 Pembroke Road, Clifton, Bristol 8. |
| *SHORE, T. L. H., M.A., M.B., B.Ch. Cantab. .. | Linden Lodge, Haines Hill, Taunton. |
| *SHORT, A. R., M.D. Lond. .. .. .. .. .. .. | 69 Pembroke Road, Clifton, Bristol 8. |
| *SHORT, E. S., M.B., Ch.B. Brist. .. .. .. | Winsley Sanatorium, near Bath. |

| | |
|---|---|
| *SILVEY, S., M.B., Ch.B. Brist. | "Denewood," Coombe Dingle, Bristol 9. |
| *SIMPSON, D., M.B., Ch.B. Glasg. | 181 Redland Road, Redland, Bristol 6. |
| *SIMPSON, T. E. N. | "West Hythe," Old Patchway, Bristol. |
| *SINCLAIR, K. S., M.D. | 25 Hill Road, Weston-super-Mare. |
| *SINCLAIR, P. S., M.D. | 520 Filton Avenue, Bristol 7. |
| *SLUGLETT, J., M.B., Ch.B. Glasg. | 2 Knowle Road, Knowle, Bristol. |
| *SMALLWOOD, A. L., M.B., B.Ch. Liverpool | The Briars, Church Road, Wick, Glos. |
| *SMART, G. A., M.D. Durh. | Bristol Royal Infirmary. |
| *SMYTHE, H. J. D., *M.C.*, M.D., M.S. Lond. | Southmead Hospital, Bristol. |
| *SNAWDON, J. W. E., M.B., Ch.B., B.D.S. Brist. | Litfield House, Clifton, Bristol 8. |
| *SOLTAU, D. K., M.A., M.B., B.Ch. Cantab. | 19 The Avenue, Clifton, Bristol 8. |
| *SOLTAU, H. K. V. | 19 The Avenue, Clifton, Bristol 8. |
| *SOMERS, MARY A. E. | 2 Ashcombe Road, Weston-super-Mare. |
| *SPARKS, J. V., B.A. Cantab. | The Middle House, Felton, Som. |
| *SPOOR, A., M.B., Ch.B. Cantab. | The Hydro, Bristol 1. |
| *STEINER, G., M.D. Vienna | 23 Manilla Road, Clifton, Bristol 8. |
| *STEPHEN, R. J. | 92 Redland Road, Bristol 6. |
| *STEVEN, G. D., M.B., Ch.B. Edin. | 6 Upper Church Street, Bath. |
| *STRUTHERS, A. J., M.B., Ch.B. Glasg. | 100 Church Road, Redfield, Bristol 5. |
| *STRUTHERS, GEO., M.B., Ch.B. Glasgow | 100 Church Road, Redfield, Bristol 5. |
| Students' Common Room, Bristol Royal Infirmary | |
| *SULLIVAN, H. W., M.B., Ch.B. | Stoke Park Colony, Stapleton, Bristol 5. |
| *SUTTON, FREDERICK, *M.C.*, M.D., F.R.C.P. | 1 Pembroke Road, Clifton, Bristol 8. |
| | |
| *TALLACK, Major R. J. K., R.A.M.C. | Medical Officer, c/o Postmaster, Zanzibar. |
| *TASKER, D. G. C., M.B., M.S. Lond. | 9 College Fields, Clifton, Bristol 8. |
| *TAYLOR, A. L., M.D. Lond. | The General Hospital, Bristol 1. |
| TAYLOR, A. L. | 35b Apsley Road, Bristol 8. |
| THIN, J. | 54 and 55 South Bridge, Edinburgh. |
| *TODD, A. T., *O.B.E.*, M.D. Edin. | Royal Park House, Clifton, Bristol 8. |
| *TOMLINSON, S., M.B., B.Ch., B.A.O. Dub. | 7 Newmarket Road, Norwich. |
| *TRIBE, WINIFRED N., M.B., Ch.B. Brist. | c/o Ebenezer Congregational Church, 10 Main Street, Ferrierastown, Johannesburg. |
| TRINICK, R. H. | 42 Cherry Valley Gardens, Belfast. |
| TRIPP, DOROTHY M. H. | Mission Hospital, Karim Nagar, Nizam's Dominions, South India. |
| *TRYON, VICTORIA S., M.B., Ch.B. Brist. | 3 Harley Place, Clifton, Bristol 8. |
| *TUDWAY, R. C., M.B., B.S., B.Sc. Lond. | Bristol General Hospital. |
| | |
| *VINTER, N. S. B., M.B., B.S. Lond. | 267 Soundwell Road, Kingswood, Bristol. |
| | |
| WAKE, S. C., M.B., Ch.B. Brist. | "Stanhope," Bideford, N. Devon. |
| *WALKER, C. H., M.B. Cantab. | 50 St. John's Road, Clifton, Bristol 8. |
| *WALKER, Prof. MILNES, M.S. Lond. | Wrington, Bristol. |
| *WALKER, W. L., M.B. | Osborne Hotel, 24 Pembroke Road, Bristol 8. |
| *WANSBROUGH, T. B., M.B., Ch.B. Brist. | 23 Pembroke Road, Clifton, Bristol 8. |
| *WARREN, R., M.A., M.D. Oxon. | Heathgates, Weston-super-Mare. |
| *WATSON-WILLIAMS, E., *M.C.*, M.B. Cantab., Ch.M. Brist. | 65 Pembroke Road, Clifton, Bristol 8. |
| WEDDELL, C. W. | Boydville, 46 Miller Street, West Melbourne, Australia. |
| *WEEKS, A. | 39 Badminton Road, Downend. |
| *WEST, H. F., M.B., B.S. Lond. | 11 Eaton Crescent, Clifton, Bristol 8. |
| *WESTHEAD, PAMELA, M.B., Ch.B. | Royal Infirmary, Bristol. |
| *WETHERED, R. R., B.A., B.M., B.Ch. Oxon | Crossways, Stoke Lane, Westbury, Bristol. |
| *WHITE, R. F., M.B., Ch.B. Brist. | "Wellclose," High Street, Nailsea. |
| *WHITTLES, J. H., M.D., B.Sc. | Cleeve Lodge, Downend, Bristol. |
| *WILBOND, R. G. | 76 Chesterfield Road, St. Andrew's Park, Bristol. |
| *WILLIAMS, I., M.B., Ch.B. Brist. | "Ardrem," Hill View, Henleaze, Bristol. |

| | |
|---|---|
| *Williams, S. R., M.B. Lond. .. .. .. .. | Buckfastleigh, Devon. |
| *Williams, P. .. .. .. .. .. .. .. .. | Chipping House, Wotton-under-Edge, Glos. |
| *Williamson, Col. M. J., *M.C.*, M.B., Ch.B. Aberd. | Rodney House, Clifton, Bristol 8. |
| *Willoughby, J., M.B., B.Ch., B.A.O. Dub. .. | "Kingsholme," Clevedon Road, Nailsea. |
| *Wohlfeld, H., M.D. Vienna .. .. .. .. | 5 Rodney Place, Clifton, Bristol 8. |
| *Wood, W. V., *M.C.*, B.A. Cantab. .. .. .. | Henley Lodge, Yatton, Som. |
| *Woodman, Dorothy, M.D. Lond. .. .. .. | 10 Tyndall's Park Road, Clifton, Bristol 8. |
| *Woods, G. E., M.B., B.S. Lond. .. .. .. | 21 Downs Cote View, Westbury, Bristol 9. |
| *Woolley, W., M.B., Ch.B. Brist. .. .. .. | 239 Cranbrook Road, Redland, Bristol 6. |
| *Woolmer, R., B.A., B.M., B.Ch. Oxon. .. | 21 Downleaze, Stoke Bishop, Bristol 9. |
| *Wright, A. J. M., M.B., B.S. Lond. .. .. | Harley Cottage, Clifton Park, Clifton, Bristol 8. |
| *Wright, Winifred M., M.B., B.S. Lond. .. | Merrymead, Shepton Mallet, Som. |
| *Zealand, L., M.D. Man. .. .. .. .. .. | Brecon Lodge, Westbury, Bristol. |

